# Incidence of Pathogenic Variants in Those With a Family History of Pancreatic Cancer

**DOI:** 10.3389/fonc.2018.00330

**Published:** 2018-08-21

**Authors:** Sarah K. Macklin, Pashtoon M. Kasi, Jessica L. Jackson, Stephanie L. Hines

**Affiliations:** ^1^Department of Clinical Genomics, Mayo Clinic, Jacksonville, FL, United States; ^2^Department of Oncology, Mayo Clinic, Jacksonville, FL, United States; ^3^Department of Diagnostic and Consultative Medicine, Mayo Clinic, Jacksonville, FL, United States

**Keywords:** genetics, hereditary cancer syndrome, pancreas cancer, family history, genetic testing

## Abstract

Discovery of a hereditary cancer syndrome can be one of the factors that determine whether a healthy individual completes pancreas cancer screening or whether an individual with cancer receives certain chemotherapies. Retrospective review was completed to determine the likelihood of detection of a pathogenic variant causing a hereditary cancer syndrome based on personal and family history. Study was completed through the hereditary cancer clinic at Mayo Clinic Florida over a 6 year period, 1/2012 through 1/2018. All participants were referred based on suspicion for a hereditary cancer syndrome based on personal and/or family history. Patients' personal oncologic history at time of consultation was recorded, as well as, cancer diagnoses in the family history and the number of family members with a history of pancreas cancer. Test result and gene name, if variant was pathogenic or likely pathogenic, were noted as well. A total of 2,019 patients completed genetic testing during study period. Personal history of cancer included a variety of primaries, including breast (*N* = 986), ovarian (*N* = 119), colon (*N* = 106), prostate (*N* = 65), and pancreas (*N* = 59). A positive result was discovered in 11% of the total group. Two hundred and eighty five reported a family history of pancreas cancer. The incidence of pathogenic variants was 13% (37/285) in those with any family history and 23% (13/56) in those with two or more relatives with pancreatic cancer. Those with multiple relatives with pancreatic cancer were significantly more likely to carry a pathogenic variant than those with a personal history of breast cancer under the age of 45 (23.2 vs. 11.9%, *p* = 0.02). Presence of multiple family members with a reported history of pancreatic cancer significantly increased the likelihood that a pathogenic variant would be identified in the patient even over other significant risk factors, like personal history of early onset breast cancer.

## Introduction

Pancreas adenocarcinoma is a heterogeneous group of diseases and can be categorized into four different subtypes: squamous, pancreatic progenitor, immunogenic and aberrantly differentiated endocrine exocrine (1). Although the median age of diagnosis is around 70 years, younger individuals in their 40s, 50s, and 60s can also develop pancreas cancer (2). The challenge of pancreas cancer remains the delay in diagnosis that occurs as a result of non-specific symptoms early in the disease process, when the tumor is most likely to be resectable (3). As a result, 80% of tumors are no longer considered to be resectable at the time of diagnosis, leading to a 5-year survival of only 8% (4). While combined modality treatments (chemotherapy and radiation) and advancement in surgeries are increasing the number of individuals who can be potentially resected, a significant number are already metastatic at the time of diagnosis and are not candidates for surgery.

Enhanced surveillance protocols may increase the likelihood of early detection and improved survival (5–7). Screening generally includes some combination of endoscopic ultrasound, magnetic resonance imaging/magnetic resonance cholangiopancreatography, computed tomography and/or abdominal ultrasound (8). A reliable serum biomarker is not available for early detection at this time (9). Caution should be used when recommending screening options as there are risks and limitations involved with screening the pancreas (10, 11). Identification of lesions through the above methods can lead to difficult decisions on how to manage findings, and the procedures themselves are not without possible complications (11, 12). Positive predictive value of pancreas screening in the general population could be <1% (10).

Some research suggests benefit from completing screening in high risk populations though such as those who have a significant family history of pancreatic cancer or a known hereditary cancer syndrome (5–7). It is estimated that genes play a significant role in around 5–15% of all pancreatic cancer diagnoses (2, 13, 14). More common related hereditary cancer syndromes include hereditary breast and ovarian cancer syndrome (HBOC), familial atypical melanoma mole-melanoma syndrome (FAMMM), Lynch syndrome and Peutz-Jegher syndrome (15).

In addition, recent developments in genetics and oncology have improved knowledge of the underlying mechanism of malignant transformation, resulting in additional treatment options of tumors (16, 17). Although systemic chemotherapeutic options for advanced pancreas cancer have not changed, there is increasing interest in identifying DNA damage-repair (DDR) and mismatch repair (MMR; Lynch Syndrome) related-pancreas cancers due to implications for patient's own treatment. MMR-deficiency, for example, can lead to a high tumor mutation burden, and there is tissue-agnostic approval of immunotherapy for this subset of tumors (16). MMR-deficiency has been noted in around 2% of the pancreas cancers tested (16). In actual practice, the prevalence is likely lower. However, given the excellent response to immunotherapy (62% response rate and 75% disease control rate) (16), Mayo Clinic FL completes MMR-deficiency screening in patients with advanced pancreas cancer.

Of more interest are the DDR-related pancreas cancers, which include the genes in the homologous recombination (HR)-pathway (e.g., *BRCA1/2)*. These tumors are more susceptible to DNA-damaging chemotherapies (classically platinum-based chemotherapies) and the newer class of agents, poly ADP-ribose polymerase (PARP) inhibitors (17). While the DDR-related tumors are seen more frequently in individuals with a prior personal or family history of breast, ovarian, prostate or pancreas cancers, these can be found in individuals without a significant history. In the rapidly growing landscape of genetics, the importance of having patients with advanced cancer, especially those with a suspicious personal or family history, seen by medical genetics for molecular testing cannot be overemphasized.

Many studies have focused on the likelihood of detection of a pathogenic variant in different populations with pancreatic cancer (18–21). In two unrelated, unselected populations of 306 and 3,030 patients with pancreatic cancer, around 5% had a positive genetic test result in both studies (18, 19). The literature is somewhat conflicting regarding the relevance of family history of pancreatic cancer. One study found that 12% of 302 patients with pancreatic cancer and a family history of pancreatic cancer carried a pathogenic variant (20). Separate research on 290 patients with pancreatic cancer showed that having a personal history or first degree relative with breast or colon cancer increased the likelihood of a pathogenic variant being detected, but a family history of pancreatic cancer did not appear to increase risk. In that study, incidences of pathogenic variants measured around 11% for personal/family history of colon or breast cancer and 3% for family history of pancreatic cancer (21). The purpose of this study was to focus more broadly on risk for a pathogenic variant based on personal and family history of pancreatic cancer. Results of individuals who completed testing as part of evaluation for a hereditary cancer syndrome due to personal and/or family history of benign and malignant tumors are reported.

## Materials and methods

This study was carried out in accordance with the recommendations of the Mayo Clinic Institutional Review Board. While this study included human subjects, it was considered exempt from Institutional Review Board approval as data was not recorded in a manner that allowed for identification of specific subjects (ID: 18-004298). Retrospective examination of the genetic test results from patients that pursued testing at Mayo Clinic Florida from January 2012 to January 2018 was completed. These patients were referred to the hereditary cancer clinic based on their personal and/or family history of cancers or tumors. All patients met with a genetic counselor or physician to complete genetic counseling before pursuing genetic testing.

Genetic testing was completed by four different, CLIA-certified commercial laboratories: Myriad Genetics, Ambry Genetics, GeneDx, and Invitae. Each laboratory would have somewhat unique processes for test methodology, variant classification, and quality control (22–25); these processes would have evolved over study time period with advances in technology and practice guidelines. As this study reports on real world data on patients who underwent testing through these four commercially available platforms, their individual methodologies were not included in this paper. The test ordered was chosen based on the discretion of the ordering provider depending on individual patient and family history and preferences of the patient. Therefore, the number of genes analyzed would vary significantly between different indications and histories provided. Genes analyzed on certain panels would also vary over time as new genetic research developed. The type of testing completed was recorded, but not genes included on each test. Details on the current list of genes tested by each of the commercially available testing platforms are available on their respective websites.

The results from the genetic testing were then logged as negative, variant of uncertain significance or positive. Variant classification was completed entirely by the genetic testing company. If a pathogenic or likely pathogenic variant was reported, the gene was noted as well. Clinical notes and a three generation pedigree taken during the original genetics consult were reviewed. An individual's personal diagnoses of cancer and the types of cancers present in the family were recorded. The likelihood of detection of a pathogenic variant in different populations was then compared. Fisher's exact test was used to compare frequency of pathogenic variants due to the small sample size of certain subsets. A *p*-value of <0.05 was considered statistically significant.

## Results

A total of 2,019 patients completed testing as part of evaluation for a hereditary cancer syndrome due to personal and/or family history of benign and malignant tumors. Patients presented with a wide array of indications, including personal history of breast (*N* = 986), ovarian (*N* = 119), colon (*N* = 106), prostate (*N* = 65), pancreatic (*N* = 59) and other cancers. A portion of the patients (154/2,019) had a history of more than one primary cancer; these included breast, ovarian, uterine, colon, renal, thyroid, bladder, adrenal cortical carcinoma, sarcoma, glioblastoma, melanoma, prostate, pancreatic, gastric, appendix, gastrointestinal stromal tumors, bile duct, leukemia, and lymphoma.

Panel testing was ordered for 1,469 (73%) of the patients. These panels ranged significantly in size depending on the date ordered and indication. The next most common tests ordered were *BRCA1/2* analysis (*N* = 395) and targeted testing for a familial variant (*N* = 115). Single gene analysis was ordered 36 times and analysis of two genes, other than *BRCA1/2*, was ordered 13 times. At least one pathogenic or likely pathogenic variant was detected in 223 (11%) patients of the total population of 2,019, with six of those having a second pathogenic variant.

Four (6.8%) of the 59 patients referred based on personal history of pancreatic cancer were discovered to have a pathogenic variant, and an apparently mosaic pathogenic variant in the *ATM* gene was detected in a fifth patient (Table 1). The patient chose not to complete additional testing that would have been necessary to better understand the nature of this variant. All patients with a pathogenic result had a family history of cancer as well. Only a minority (*N* = 5) of those tested with a personal history of pancreatic cancer had no family history of breast, pancreatic, colon or ovarian cancer though.

**Table 1:** Incidence of pathogenic variants in different populations.

**Category**	**Positives/Total (%)**	**Genes**
Personal history of pancreatic cancer	4–5/59 (7–8%)	*ATM*^a^, *ATM, BRCA1* (2), *BRCA2*
Personal history of breast cancer	81/986 (8%)	*ATM* (4), [*ATM*/*CHEK2*]^b^, *ATM*^a^, *BRCA1* (16), *BRCA2* (26), [*BRCA2*/*CHEK2*]^b^, [*CHEK2*/*PALB2*]^b^, *BARD1, CHEK2* (14), *CDKN2A, MSH6* (2), *NBN, PALB2* (8), *PMS2, TP53* (2)
Personal history of breast cancer diagnosed ≤ age 50	47/486 (10%)	*ATM* (3), [*ATM*/*CHEK2*]^b^, *BRCA1* (9), *BRCA2* (15), *BARD1, CHEK2* (10), *CDKN2A, PALB2* (4), [*PALB2*/*CHEK2*]^b^, *TP53* (2)
Personal history of prostate cancer	9/65 (14%)	*APC*^c^, *ATM, BRCA1, BRCA2* (2), *CHEK2* (2), *MLH1, TP53*
Personal history of colon cancer	14/106 (13%)	*APC, BRCA1* (2), [*BRCA2*/*MUTYH*]^b^, *CHEK2, EPCAM, MLH1* (3), *MSH2, MSH6, MUTYH, PMS2, TP53*
Personal history of ovarian cancer	10/119 (8%)	*ATM, BRCA2* (6), [*BRCA2*/*NBN*]^b^, *BRIP1, NBN*
Personal history of multiple primary cancers^d^	21/154 (14%)	*ATM*^a^, *ATM, BRCA1* (3), *BRCA2* (6), [*BRCA2, MUTYH]*^b^*, CDKN2A, CHEK2* (4), *MLH1, MSH6, NBN, TP53*
Family history of pancreatic cancer	37/285 (13%)	*APC*^c^ (3), *ATM* (5), *BRCA1* (4), [*BRCA1/CDKN2A*]*^*b*^, BRCA2* (13), *CHEK2* (5), *MSH6, NBN, PALB2* (3), *PRSS1*
Family history of 2+ relatives with pancreatic cancer	13/56 (23%)	*APC*^c^ (2), *ATM* (3), *BRCA1* (2), *BRCA2* (4), *CHEK2* (2)

a *Apparently mosaic pathogenic variant*.

b *Two pathogenic variants detected simultaneously*.

c *APC Ashkenazi Jewish founder mutation*.

d *Primary cancers included breast, ovarian, uterine, colon, renal, thyroid, bladder, appendix, gastrointestinal stromal tumors, adrenal cortical carcinoma, sarcoma, glioblastoma, melanoma, prostate, pancreatic, gastric, bile duct, leukemia, and lymphoma*.

Two hundred and eighty-five patients reported a family history of pancreatic cancer. This was not limited to only first and second degree relatives. About 1/3 (*N* = 91) of those with a family history of pancreatic cancer had a personal history of a suspicious cancer or tumor. A pathogenic variant was detected in 37 (13.0%). Fourteen of the 37 had no personal history of cancer or tumors. If two or more relatives had been diagnosed with pancreatic cancer, the incidence of pathogenic variants rose to 23.2% (13/56). Individuals with at least two relatives with pancreatic cancer were statistically more likely to have a pathogenic variant identified compared to all those with a personal history of breast cancer ≤age 50 (23.2 vs. 9.7%, *p* = 0.006) and ≤age 45 (23.2 vs. 11.9%, *p* = 0.02).

Of the patients with a personal or family history of pancreatic cancer and a pathogenic result in a hereditary breast cancer gene, around 40% (15/35) did not meet Medicare criteria for HBOC testing. The majority of those (13/15) missed Medicare criteria due to lack of personal history of breast, ovarian/fallopian tube, pancreatic or prostate cancer. Mean age of those that did not meet criteria was 52.3 (SD: 14.9), and 6/15 had a known familial mutation.

## Discussion

The results of this study show the importance of obtaining detailed family history while evaluating a patient. A significant number of those with a reported family history of pancreatic cancer were discovered to carry a pathogenic variant regardless of degree of relation. Those with at least two relatives diagnosed with pancreatic cancer were significantly more likely to carry a pathogenic variant than those with a personal history of breast cancer diagnosed at or under the age of 45. The National Comprehensive Cancer Network (NCCN) recommends genetic risk evaluation for anyone diagnosed at 50 or under with breast cancer (27), and Medicare supports testing for anyone diagnosed at age 45 or under with breast cancer. In our study, many of those with a pathogenic HBOC variant and family history of pancreatic cancer would not have met Medicare criteria for testing. Other research has shown similar results; 6 of the 14 people that tested positive for a pathogenic *BRCA1/2* in another pancreas cancer study did not meet the NCCN's criteria for germline testing (18). Medicare criteria supports HBOC testing for individuals with a personal history of pancreatic cancer if they have Ashkenazi Jewish ancestry, one close relative with breast cancer at age 50 or under, one close relative with ovarian cancer at any age, or at least two close relatives with breast, pancreatic or prostate cancer. Medicare will not cover HBOC genetic testing if the patient has not personally been diagnosed with breast, pancreatic, prostate or ovarian cancer. The guidelines for testing vary for indication among insurance companies, but some individuals with germline pathogenic variants can be missed. This has implications not only for that individual but also for the individual's family.

The International Cancer of the Pancreas Consortium (CAPS) provided guidance on which individuals should be considered candidates for pancreas screening (8). Those with a pathogenic variant in *CDKN2A, BRCA2, PALB2*, or a Lynch syndrome-associated gene and a first degree relative are potential candidates for screening (8). Adults with a pathogenic *BRCA2* variant and at least two relatives with pancreatic cancer and all patients with Peutz-Jegher syndrome also meet high-risk criteria (8). The CAPS guidelines do not recommend any surveillance for those with a family history of pancreatic cancer and a pathogenic *BRCA1* mutation. In this study, more individuals with a family history of pancreatic cancer had a pathogenic *BRCA2* variant, but more of those with a personal history of pancreatic cancer had a *BRCA1* variant. This is likely related to small sample size, but is an interesting observation as typically in larger cohorts, the risk of pancreas cancer is higher in the *BRCA2* cohort (26).

Identification of a germline and/or somatic mutation can be important for those already diagnosed with advanced pancreatic cancer as well. Patients with pancreatic cancer and a germline *BRCA1/2* mutation have more favorable outcomes and may respond better to platinum chemotherapies and PARP inhibitors (28, 29). The list of genes in the HR-pathway, which can lead to DDR-related pancreas cancer, is increasingly being recognized and explored in many clinical trials across the country and worldwide. O'Reilly and colleagues published the results of a phase-1 trial combining a platinum chemotherapy with a PARP inhibitor and showed an objective response rate of 77.8% and a median overall survival of 23.3 months (17). Compared to the median overall survival of ~9 and 11 months with Gemcitabine with Nab-Paclitaxel, and FOLFIRINOX, this result appears very promising (30, 31). Similarly, confirming whether a patient has Lynch syndrome has significant implications as immunotherapy can then be utilized during treatment (16).

Ongoing research on larger cohorts of pancreas cancer patients or high-risk groups and families are showing similar trends and numbers in terms of a 5–15% presence of DDR-related tumors (2, 13, 14). This suggests consideration of expanding the recommendation on which patients with pancreatic cancer should complete germline testing. This population provides insight from a working clinic with common practice variables, like laboratory variances and testing variability, based on patient needs and expansion in knowledge over time. Further research should be completed on larger populations tested with a standardized list of genes to expand and confirm these preliminary findings. It is important to keep in mind that all family history was self-reported, which has its limitations (32–34). The reported pancreatic cancers in relatives cannot be confirmed to have been adenocarcinomas, neuroendocrine tumors, etc. Also, all of these patients were referred to a genetics clinic based on suspicion for a hereditary syndrome. It would be helpful to further study incidence of pathogenic variants in those with family history of pancreatic cancer in different populations. With the exciting recent research findings regarding outcomes for those with DDR and MMR-related tumors, it is more important than before to identify those with relevant germline variants (16, 17).

## Author contributions

SM, PK, JJ, and SH contributed to concept and project planning. SM, PK, and SH contributed significantly to the writing of the paper. JJ contributed significantly through editing of final product.

### Conflict of interest statement

The authors declare that the research was conducted in the absence of any commercial or financial relationships that could be construed as a potential conflict of interest.
